# Multiscale bone quality analysis in osteoarthritic knee joints reveal a role of the mechanosensory osteocyte network in osteophytes

**DOI:** 10.1038/s41598-019-57303-z

**Published:** 2020-01-20

**Authors:** Gustavo Davi Rabelo, Annika vom Scheidt, Felix Klebig, Haniyeh Hemmatian, Mustafa Citak, Michael Amling, Björn Busse, Katharina Jähn

**Affiliations:** 1 Department of Osteology and Biomechanics, University Medical Center Hamburg-Eppendorf, Hamburg, Germany; 2 Helios-ENDO Klinik Hamburg, Hamburg, Germany

**Keywords:** Bone, Diseases

## Abstract

Osteophytes - bony outgrowths on joint structures - are found in healthy individuals but are specifically present in late osteoarthritis (OA). Osteophyte development and function is not well understood, yet biomechanical stimuli are thought to be critical. Bone adapts to mechanical forces via the cellular network of osteocytes. The involvement of osteocytes in osteophyte formation and maturation has not been unravelled. Forty-three osteophytes from tibias of 23 OA patients (65 ± 9 years) were analysed. The trabecular bone structure of osteophytes presented with fewer trabeculae of lower bone mineral density compared to subchondral bone. We identified 40% early stage and 60% late stage osteophytes that significantly differed in their trabecular bone characteristics. Osteophyte bone revealed a higher number of osteocytes and a lower number of empty osteocyte lacunae per bone area than the subchondral bone. We found that OA osteophytes consist of younger bone material comprised of woven and lamellar bone with the capacity to develop into a late stage osteophyte potentially via the involvement of the osteocyte network. Our analysis of OA osteophytes implies a transition from woven to lamellar bone as in physiological bone growth within a pathological joint. Therefore, osteophyte development and growth present a valuable research subject when aiming to investigate the osteogenic signalling cascade.

## Introduction

Osteophytes are macroscopically sized bony outgrowths that can form on any bone surface but are prone to form within joint structures^1,2^. The formation of osteophytes is common and present in different joints e.g. knee, hip, vertebra^3–5^. Known risk factors include but are not limited to advanced age, inflammatory pathologies, mechanically induced joint instability, but also heavy physical activity^4^. Therefore, (*i*) osteophytes present within the vertebral column of osteoporotic patients and locally counteract the general bone loss with osteoporosis, (*ii*) osteophytes also form within rheumatoid arthritic joint structures supported by or persisting the high inflammatory load, (*iii*) osteophytes are located within pathologically degenerated joints of osteoarthritic patients whereby joint instability or mechanical joint damage are present, and *iv)* osteophytes can be found in joints of healthy people^6–8^. Based on the discrepancy of osteophyte formation in health and disease, their general role in bone homeostasis and potential impact on therapeutic developments remains to be determined.

Osteoarthritis (OA) is a progressive disorder of joints affecting about 11% of the population over 60 years of age. Current pathophysiological concepts acknowledge OA as a disease of the whole joint. Changes in the subchondral bone are a hallmark in the progression of OA and bone formation is locally imbalanced leading to the growth of osteophytes^9,10^. In OA, osteophyte formation is of clinical significance: Later stage osteophytes are suggested as a source of pain. They may also limit joint mobility and contribute to a loss of function, once their growth culminates with limited movement^11^. With disease progression, an increase in local joint space narrowing is associated with an increasing osteophyte size. Early osteophyte growth (osteophyte grade 1) is mostly directed outwards creating osteophytes parallel to the main joint surfaces, potentially stabilizing the joint due to a bigger surface. With osteophyte growth, OA progression, and joint space narrowing, the osteophytes undergo a directional change from parallel to perpendicular in respect to the main joint surface (osteophyte grade 2 and 3)^12^. With continuing growth, osteophytes can limit the range of motion for the patient in the later stage of OA (Kellgren-Lawrence stage 3–4). Specifically, osteophytes develop in response to joint instability^13–15^ and have been interpreted as an attempt to counteract the effects of osteoarthritic lesions^16^. Within this line of thought, it has been theorized that osteophytes form initially to stabilize the OA joint^17^. Several studies attempt to support the hypothesis: (*i*) The removal of osteophytes in humans subjected to total knee arthroplasty led to an increase in varus–valgus joint motion^18^. (*ii*) Dayal *et al*. reported a reduced laxity in anterior posterior with osteophyte growth^19^. (*iii*) In a mouse model following anterior cruciate ligament transection, the initial increase in joint laxity was reduced significantly over time and this development correlated with the formation of osteophytes^20^. Yet, a clinical study to confirm improvement of OA-related pain and immobilization during early disease stages is still lacking. Furthermore, from a mechanical point of view, the expansion of the articular surface due to early osteophytes should theoretically lead to lower local joint stresses: Stresses are defined as a force divided by the loaded area; with a constant force (e.g. body weight in one-legged stance) an increase of the loaded area (joint surface + osteophytes) would reduce the absolute local stresses on the joint surface, which could be relevant for joint degradation. Finally, the question remains if osteophytes develop as a physiological adaptation to changes in mechanical forces or require a pathologically altered joint milieu for their formation.

The potential of bone to adapt to mechanical stimuli has been known for decades^21^. Osteocytes, with their extensive cellular network, have emerged as bone’s mechanosensory cells^22^. During new bone formation some osteoblasts are encased by osteoid and differentiate to dendritic osteocytes that end up within a three-dimensional fluid-filled lacuno-canalicular system, which spans through the entire bone matrix. Osteocytes regulate bone turnover by acting on bone-forming osteoblasts and bone-resorbing osteoclasts^23^. Moreover, osteocytes directly participate in osteolysis^24,25^, control phosphate homeostasis^26^, and functionally act on the neighbouring skeletal muscle^27^ and distant organs^28^. Therefore, the integrity of the osteocyte network is important for healthy bone tissue. Osteocyte cell death resulting in empty or even mineralized lacunae can be a dramatic endpoint of the network disruption^29^ and is a common feature of bone pathologies, e.g. osteoporosis^30^. With osteophytes being involved and potentially originating through altered mechanosensation, a functional role of the osteocyte network within these bony spurs would be likely.

We have analysed osteophytes formed in osteoarthritic knee joints. The *ex vivo* material was assessed by micro-computed tomography to allow for macro- and micro-characterization of the individual osteophyte structures. Histological analyses enabled the qualitative tissue analysis and quantitative assessment of osteocyte network characteristics. Combining these multi-scale methods, we aim to provide evidence (i) that osteophytes possess a bone composition resembling young bone matrix and arise from an active remodelling process, (ii) that the osteocyte network differs significantly within osteophytes compared to adjacent bone, (iii) that the alterations in tissue composition and osteocyte network characteristics are dependent upon osteophyte maturation, suggesting an adaptive process.

## Results

### Histomorphometry reveals distinct differences between osteophytes and the adjacent subchondral bone

**Figure 1:**
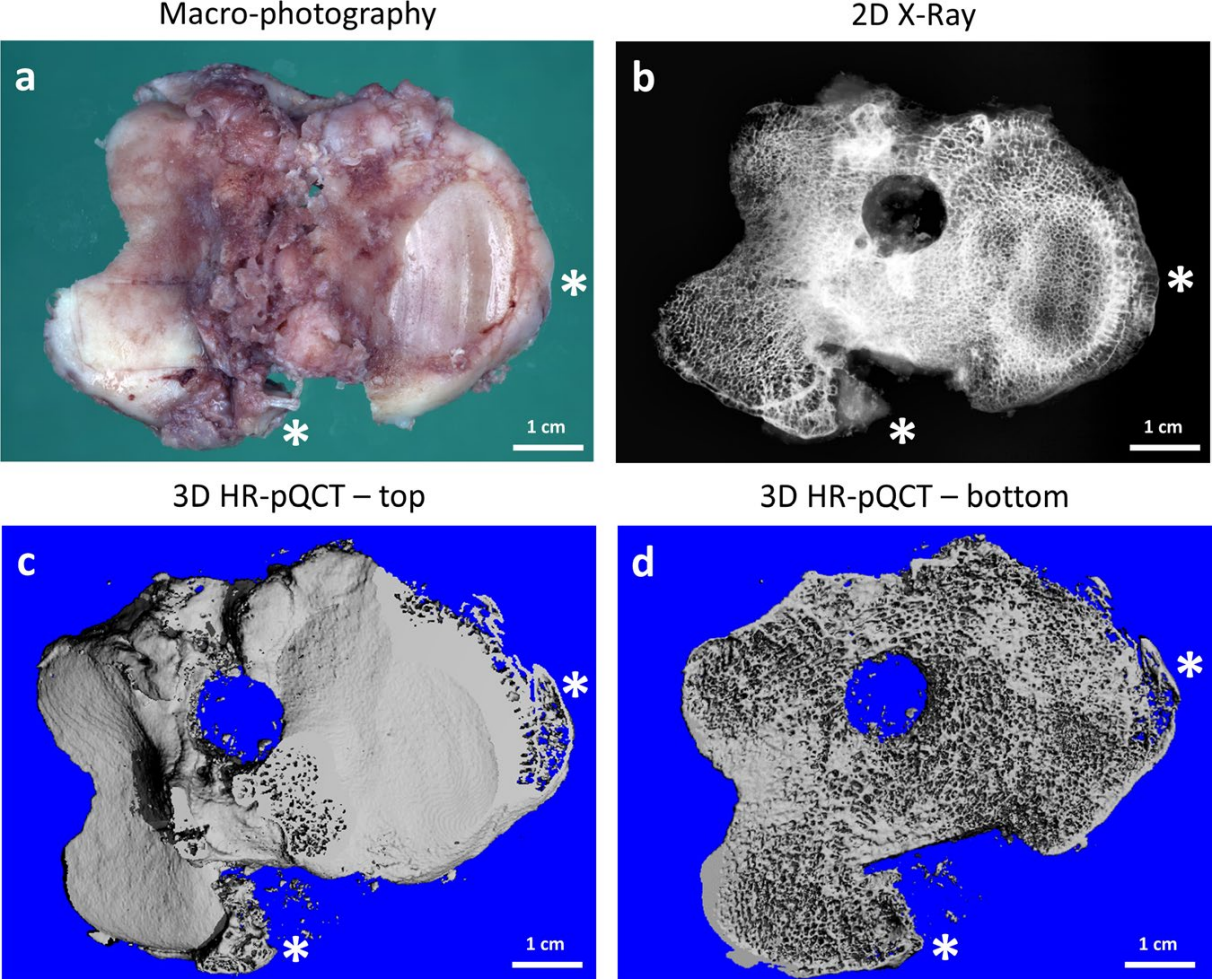
Macroscopic evaluation of a representative tibia plateau specimen. (**a**) Photography showing two osteophyte regions (*). (**b**) X-ray imaging revealing the gross bone structural arrangement. 3D reconstruction of the HR-pQCT analysis – top view of joint surface (**c**) and bottom view (**d**) of the specimen.

Osteophytes were visible on the joint surface of the specimens (Brightfield imaging: Fig. 1a, two-dimensional (2D) X-ray imaging: Fig. 1b, three-dimensional (3D) X-ray imaging: Fig. 1c,d).

**Figure 2:**
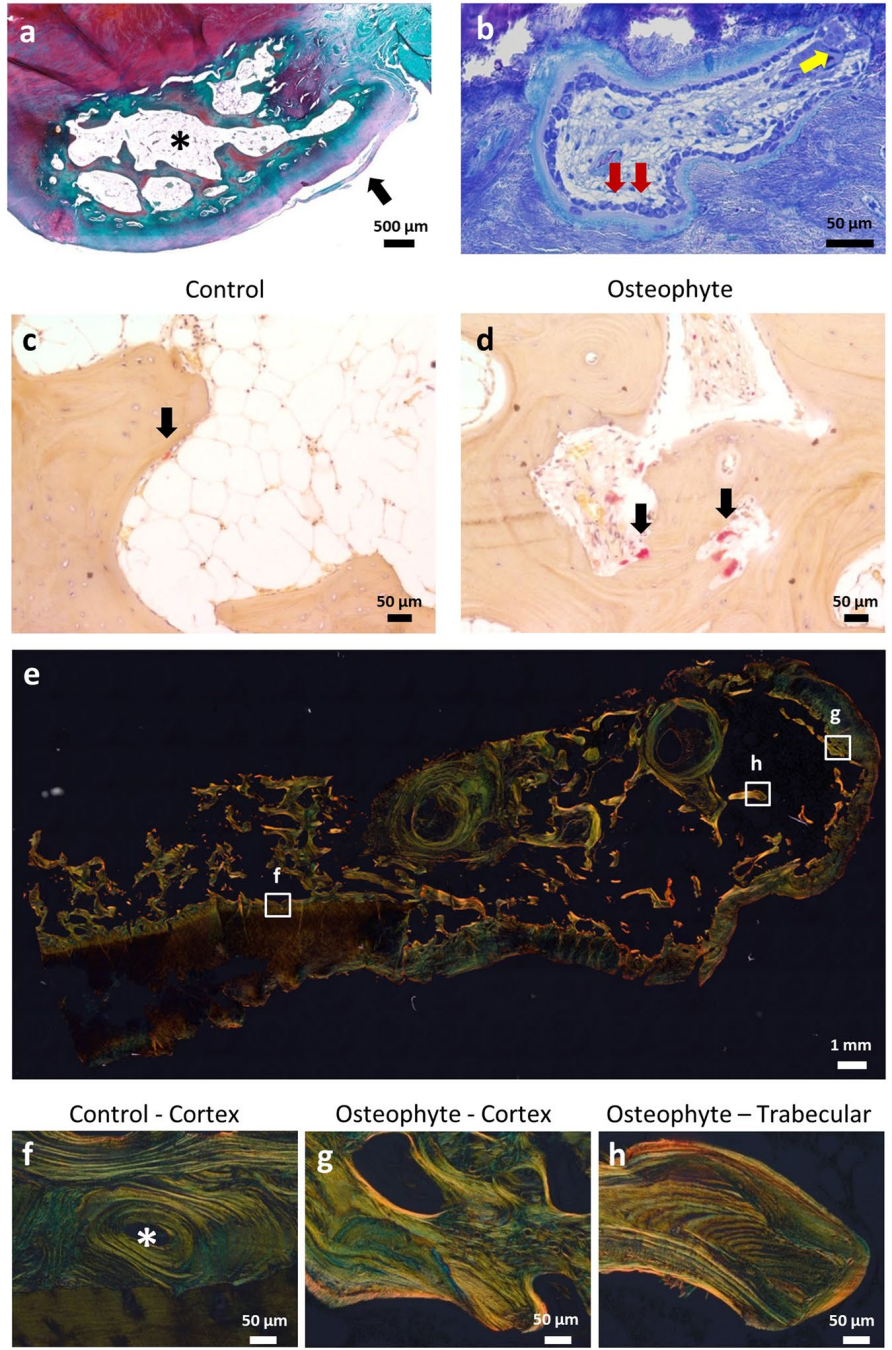
Histological assessment of the osteophyte. (**a**) Osteophyte panoramic view (decalcified, Safranin O, fast green) showing the presence of a fibrous layer (black arrow) covering the osteophyte (*). (**b**) High bone turnover, with bone formation by numerous osteoblasts (red arrows) on top of a thick osteoid layer, and the presence of osteoclasts (yellow arrow; non-decalcified, toluidine blue). TRAP staining of osteoclasts, (**c**) subchondral bone presenting with less bone resorbing osteoclasts then (**d**) osteophyte. (**e**) Polarized light microscopy mosaic image of osteophyte containing sample. (**f**) Lamellar collagen organization within an osteon (*) of a non-osteophyte cortex, (**g**) Collagen organization of the osteophyte surface, and (**h**) the osteophyte trabecular bone (non-decalcified, Picrosirius red).

Solely the osteophyte-related joint surface was covered by a fibrous tissue layer (Fig. 2a) that was variable in its thickness, with some layers being as thick as 300 µm (Supplemental Fig. 1a–c). The loss of functional cartilage is a hallmark of osteoarthritis and we found that in accordance, the hyaline joint cartilage had been degraded. In contrast, most of the osteophytes were covered by a thick cartilage layer (Supplemental Fig. 1b). Moreover, the chondrocytes were often found to form cell clusters within the cartilage (osteophyte: Supplemental Fig. 1e, control sample subchondral bone: Supplemental Fig. 1d). Subchondral cysts were determined in about 25% of the specimens.

Within the trabecular bone of the osteophytes most bone surfaces were covered with cuboidal osteoblasts producing large amounts of newly formed, unmineralized bone matrix - osteoid (Fig. 2b). This indication of highly active bone formation in osteophyte bone contrasts with the markedly low number of osteoblasts in the subchondral bone. Also, exclusively in osteophytes, there were osteoclasts within their resorption pits (Fig. 2b). Using tartrate resistant acid phosphatase (TRAP) activity staining, we detected more bone resorbing osteoclasts within osteophytes (Fig. 2c,d). Clearly, our results point to a high bone turnover activity in osteophytes.

Utilizing circularly polarized light microscopy, the collagen in cortical bone revealed a different organization in the presence or absence of osteophyte formation (Fig. 2e–h). A typical osteonal, lamellar collagen arrangement was only seen in the original cortex (alternating bright and dark lines, Fig. 2f), whereas the cortex of the osteophyte showed no clear lamellar orientation (Fig. 2g). In contrast, the trabecular bone of the osteophyte displayed a lamellar bone collagen orientation in the trabecular bone packets (Fig. 2h).

### Trabecular bone architecture within osteophytes is significantly altered

**Figure 3:**
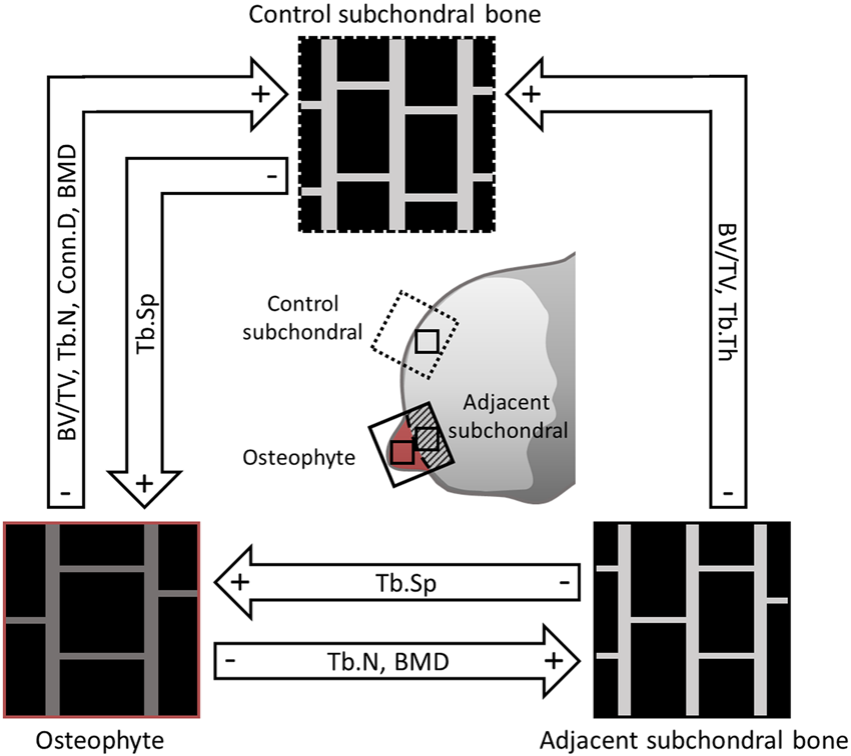
Schematic overview of µCT results and origin of specimens. Trabecular microstructure varies between osteophyte bone, adjacent subchondral bone and control subchondral bone.

Trabecular bone structure analysis revealed significant differences (Table 1, Fig. 3). Here, osteophyte bone and the adjacent subchondral bone presented a lower bone volume compared to the distant control subchondral bone (BV/TV, p < 0.0001). Trabecular connectivity was lower in osteophytes compared to the control subchondral bone, (Conn.D, p = 0.024). While SMI analysis pointed to a more rod-shaped trabecular structure in osteophytes and adjacent subchondral bone compared to the control subchondral bone (SMI, p < 0.0001), this result has to be interpreted with caution as SMI is influenced by BV/TV^31^. Osteophytes also showed a lower number of trabeculae compared to the control subchondral bone (Tb.N, p = 0.0003). Further, these fewer trabeculae were thinner than the ones in the control subchondral bone, resulting in a larger trabecular separation occurring in osteophytes compared to both subchondral areas (Tb.Sp, p < 0.0001). The mineral density in osteophyte trabeculae was lower compared to both the subchondral bone and the control area (BMD, p < 0.0001).

**Table 1:** Trabecular bone parameters in osteophytes are different from neighbouring bone (ANOVA with Tukey post-hoc test, p < 0.05, a > b).

Parameter	Control area	Subchondral bone	Osteophyte	p-value
BV/TV [%]	0.32 ± 0.03^**a**^	0.22 ± 0.01^**b**^	0.18 ± 0.01^**b**^	<0.0001*
Conn.D	20.16 ± 3.24^**a**^	15.21 ± 2.00^**a,b**^	13.90 ± 1.66^**b**^	0.024*
SMI	0.83 ± 0.13^**b**^	1.40 ± 0.09^**a**^	1.64 ± 0.09^**a**^	<0.0001*
DA	1.41 ± 0.05	1.37 ± 0.03	1.33 ± 0.02	0.15
Tb.N [/mm]	3.17 ± 0.31^**a**^	2.67 ± 0.19^**a**^	1.97 ± 0.11^**b**^	0.0003*
Tb.Th [µm]	158.96 ± 6.27^**a**^	139.99 ± 4.60^**b**^	140.98 ± 4.60^**a,b**^	0.038*
Tb.Sp [µm]	416.31 ± 33.50^**b**^	466.34 ± 22.21^**b**^	600.37 ± 26.94^**a**^	<0.0001*
BMD [mg HA/m^3^]	829.07 ± 7.38^**a**^	820.00 ± 5.21^**a**^	792.13 ± 4.88^**b**^	<0.0001*

### Higher number of viable osteocytes in osteophytes compared to adjacent subchondral bone

**Figure 4:**
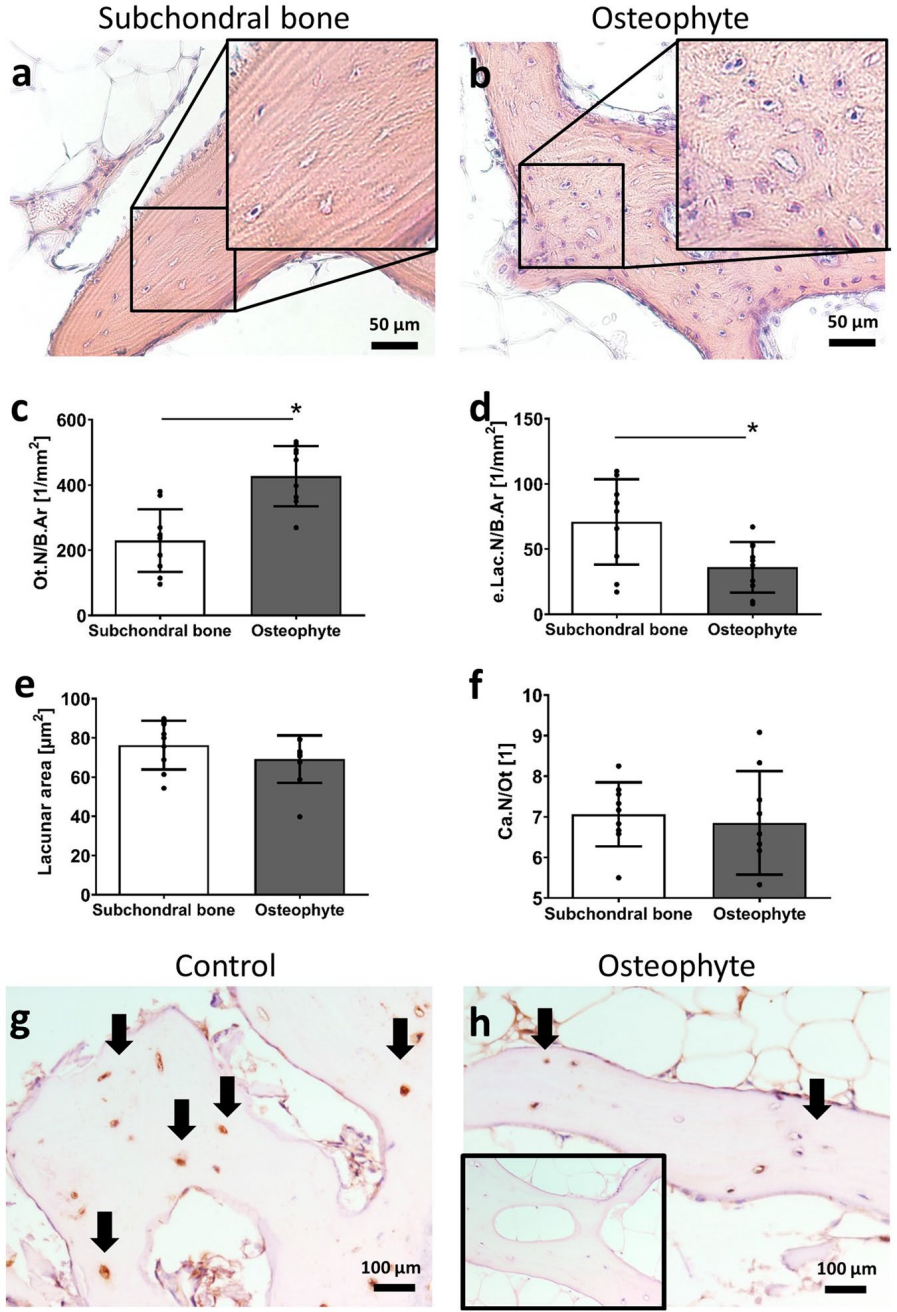
Osteocyte network characteristics within osteophytes. (**a**,**b**) representative images of osteocytes and their lacunae (decalcified, H&E, insert magnified 2×). Bar graphs show (**c**) mean osteocyte number per bone area (p < 0.0001), (**d**) number of empty lacunae (p = 0.003), (**e**) lacunar area, and (**f**) canalicular number per osteocyte. Paired t-test; p < 0.05. Sclerostin immunohistochemistry showing (**g**) many, dark stained osteocytes in non-osteophyte bone and (**h**) fewer, weaker stained osteocytes in osteophyte bone. (**h, insert**) Negative control without primary antibody.

Representative images indicate the presence of more osteocytes in osteophytes compared to subchondral bone (Fig. 4a,b). The osteophyte bone presented with a significantly larger number of osteocytes compared to the adjacent subchondral bone (Fig. 4c, Ot.N/B.Ar, p < 0.0001). In addition, osteophytes had lower empty osteocyte lacunae than the adjacent subchondral bone (Fig. 4d, e.Lac.N/B.Ar, p = 0.003). Surprisingly, in some of the osteophytes it was possible to identify osteocyte lacunae with two cell nuclei in one lacuna (Supplemental Fig. 2a). Following quantification, two cell nuclei in one lacuna occurred significantly more often in osteophytes in comparison to adjacent subchondral bone (4.34 ± 0.83 vs. 1.41 ± 0.67, p = 0.039). Both the lacunar area and the number of canalicular processes (exemplary images in Supplemental Fig. 2b) showed no significant differences between the groups (Fig. 4e,f, Lacunar area, Ca.N/Ot).

With osteocytes being the mechanosensors of the bone tissue, the osteocyte product sclerostin is a potent inhibitor of bone formation in situations of unloading^32^. We detected a less pronounced immunostaining of sclerostin within osteophytes compared to the adjacent bone and the distant subchondral bone (representative images: distant: Fig. 4g, osteophyte: Fig. 4h). The lower sclerostin expression was associated with fewer and weaker labelled osteocytes in osteophytes.

### Trabecular bone differences relate to maturation stages of the osteophytes

**Figure 5:**
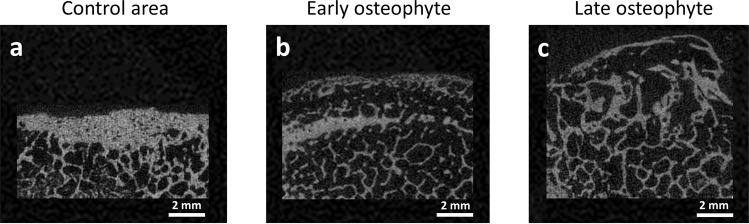
Representative images of osteophyte stages and control area (µCT scan). (**a**) Control area, (**b**) early stage and (**c**) late stage osteophyte.

In accordance with Wong *et al*. osteophytes were divided into early stage and late stage osteophyte (bone without osteophyte: Fig. 5a, early stage: Fig. 5b and late stage: Fig. 5c)^4^. We determined about 40% of early and 60% of late stage osteophytes. Defined trabecular bone characteristics were significantly different between these groups (Table 2). Early stage osteophytes were smaller than late stage ones (TV, p = 0.016). The trabecular connectivity density in early osteophytes suggested more structural connection than in late osteophytes (Conn.D, p = 0.058) and early osteophytes had more trabeculae with less separation than late ones (Tb.Th, Tb.N, p ≤ 0.011).

**Table 2:** Distinct differences in trabecular bone parameters of early and late osteophytes.

Parameter	Early osteophyte	Late osteophyte	p-value
TV [mm³]	49.00 ± 9.88	113.74 ± 19.22	0.016*
BV/TV [%]	0.20 ± 0.02	0.17 ± 0.01	0.324
Conn.D	17.27 ± 3.27	11.20 ± 1.21	*0.058*
SMI	1.76 ± 0.16	1.48 ± 0.10	0.136
DA	1.33 ± 0.04	1.34 ± 0.02	0.668
Tb.N [/mm]	2.31 ± 0.20	1.72 ± 0.08	0.004*
Tb.Th [µm]	139.38 ± 7.11	143.78 ± 5.87	0.65
Tb.Sp [µm]	524.09 ± 38.83	655.13 ± 29.33	0.011*

### Adaptation of the mechanosensory osteocyte network in early vs. late osteophytes

**Figure 6:**
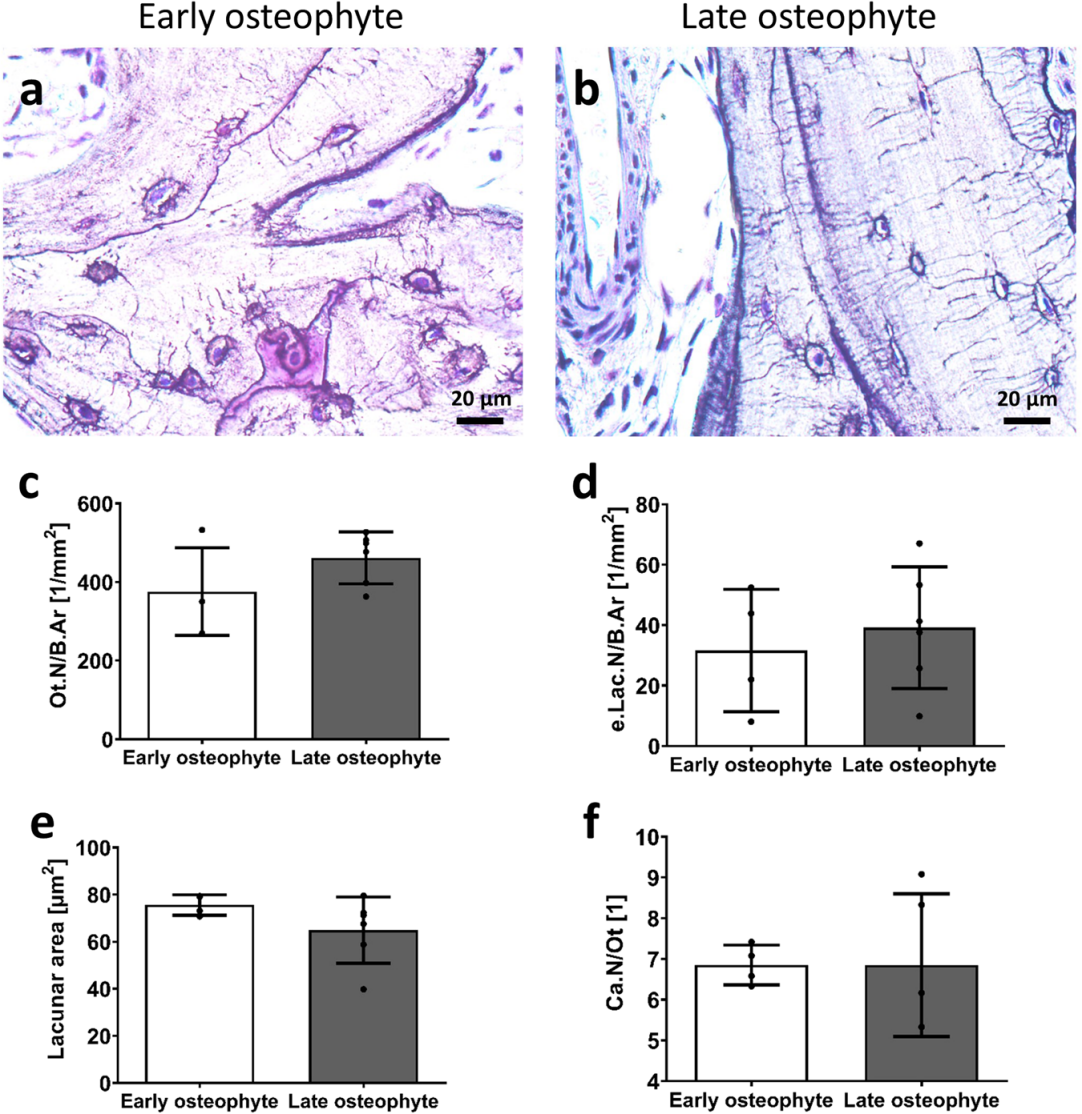
Comparison of the osteocytes network in early stage and late stage osteophytes. (**a**,**b**) representative images (decalcified, silver precipitation). Lacunae with connected canaliculi including the blue labelled osteocyte nuclei can be seen in the osteophyte trabecular bone. Bar graphs show (**c**) mean osteocyte number per bone area, (**d**) number of empty lacunae, (**e**) lacunar area, (**f**) canalicular number per osteocyte. Unpaired t-test with p < 0.05.

Early stage osteophytes appeared to have larger lacunae and less numerous osteocytes compared to late stage osteophytes (Fig. 6a,b). If quantified, the highest number in viable osteocytes was detected in late osteophytes compared to early ones, but this difference did not reach statistical significance (Fig. 6c, p = 0.160). The number of empty lacunae was variable and no significant difference between the groups was determined (Fig. 6d, p = 0.578). Yet, the phenomenon of double nuclei within one lacuna was different between early and late osteophytes (5.87 ± 0.87 vs. 2.18 ± 0.50, p = 0.0048) suggesting a time-dependent influence. While the lacunar area tended on average to be larger in early osteophytes compared to later ones, significance was not reached (Fig. 6e, p = 0.184). Finally, canalicular number per osteocyte did not change between the groups (Fig. 6f).

## Discussion

In this study, we determined that osteophytes in human osteoarthritic knee joints possessed a bone matrix quality different from the adjacent subchondral and the distant subchondral bone tissue. The osteophyte bone was characterized by a less mature bone matrix as determined by µCT BMD in trabecular bone, as well as polarized light microscopy for collagen orientation in cortical bone. We found signs of high bone turnover in a collective of osteoarthritic knee osteophytes and determined by µCT that bone tissue quality in osteophytes was being altered during a maturation process. Finally, the osteocyte network in osteophytes varied from the one found in the adjacent subchondral bone with a higher number of viable cells and lower sclerostin expression, and underwent slight changes during osteophyte maturation, suggesting a role for mechanotransduction with osteophyte formation and growth.

We determined differences between bone microarchitecture and density in osteophytes, adjacent subchondral and distant subchondral bone. Compared to both adjacent and distant subchondral bone, osteophyte bone showed a lower bone mineral density. This lower bone mineral density demonstrates a younger tissue age for osteophyte bone compared to subchondral bone as increased mineral content is associated with tissue aging^33,34^.

In the OA-affected joint, control subchondral bone showed a higher trabecular bone volume compared to the bone microstructure within osteophytes and within osteophyte-adjacent subchondral bone. The higher trabecular bone volume fraction found in control subchondral bone indicates an adaptation to higher loads occurring in the control subchondral bone in comparison to loads occurring in the osteophyte bone. In addition, osteophyte microstructure showed a lower trabecular number and connectivity compared to control subchondral bone. These findings could support the hypothesis that during their maturation, osteophytes may grow to increase the joint surface^11^ and thereby distribute the total load over a larger surface, reducing local stresses. The osteophyte-adjacent subchondral bone had not only a lower BV/TV but also thinner trabeculae. While our study did not allow analysing the development of the bone microstructure over time directly, other studies have shown trabecular thinning to be a common bone resorption mechanism^35^. It is possible that some of the observed differences in microstructure are related to bone resorption driven by a beginning joint immobilization. The latter can be seen in patients due to increasing OA-related joint pain.

While our analysis showed osteophytes and the subchondral bone to have more rod-shaped trabeculae compared with subchondral bone in the control area, this is based on SMI and has to be interpreted with caution as Salmon *et al*. reported a strong correlation between SMI and BV/TV, often rendering rod/plate interpretations incorrect^31^.

In addition to the observed lower BMD, we determined a larger amount of osteoid, active osteoblasts and active osteoclasts in osteophytes, suggesting an elevated remodelling activity that could contribute to further development of the osteophyte structure. Also, our previous work revealed the presence of linear microcracks - a target for bone remodelling - within osteophytes^5^. When these microcracks were present in the osteophyte area, the microdamage density was also significantly higher in the whole femoral neck in hip OA^5^. Interestingly, we found the cortical surface of the osteophyte to be more heterogeneous and non-lamellar in its collagen organization, while the cortices of neighbouring subchondral regions revealed a typical lamellar organization and the presence of osteons. To our knowledge, there was no previous information regarding the lamellarity and we have noted that this lamellar arrangement was rare in our cohort. Changes in cartilage integrity and a surface fibrous tissue layer were also detected. As in accordance with Junker *et al*. we found a mesenchymal connective tissue cap on the upper surface of the osteophytes^2^. Within our study, the fibrous tissue covered a cartilage layer, supported by a non-lamellarly organized cortex enveloping a trabecular bone volume. Similar aspects, including active remodeling zones on the bone surfaces, an attached cartilage layer, and the presence of marrow spaces in between the trabecular bone of osteophytes were previously described^2^. Therefore, we would like to propose the following possible sequence of events: Due to cartilage damage the subchondral bone experiences very high local stresses and due to these very high local stresses the osteophyte forms. The resulting joint surface of original and osteophyte surface is larger and leads to a lower local stress under an assumed constant total load. Following the initial growth, the osteophytes transitions from woven bone to lamellar bone in a similar manner as in normal bone development and may continue to grow. During the initial growth of the osteophyte the adjacent subchondral bone adapts to the lowered local stress.

We then aimed to determine differences in osteophyte maturation to reveal a potential time-dependent adaptation process. We noted that late stage osteophytes were larger with fewer trabeculae and greater trabecular separation. The higher number of trabeculae in early osteophytes indicate a higher bone deposition during osteophyte formation. We hypothesize that the mechanical stimulus on the osteophyte is stronger and consequently more anabolic during this stage. This could be further explained by the smaller size of the early osteophytes: With a smaller joint surface extension, the local stress should be higher compared to a larger, late stage osteophyte that could distribute the load over a wider surface. The ongoing remodelling process during osteophyte maturation leads to a disappearance of the original cortex together with bone formation to incorporate the osteophyte trabecular bone into the one of the adjacent subchondral bone. This process could support the notion that osteophytes form and adapt to mechanical strains in the joint environment while also being associated with joint immobilization and pain. Oni and Morrison revealed that force-deformation curves from mechanical tests of knee osteophytes were heterogeneous and with a marked variability between specimens^36^. As the cartilage covering the osteophyte was mechanically inferior to normal articular cartilage, they concluded that this presumably reflects differing loading requirements at these sites. We detected the presence of a mechano-regulated inhibitor of bone formation – sclerostin^37,38^ – in osteocytes of our specimens. Here, labelling was more pronounced in the subchondral bone than in osteophytes. This result is in line with a lower formation activity in subchondral bone due to unloading via the structural support of the osteophyte and endorses the assumption that osteophyte formation is being induced mechanically. Overall, the sensation of the mechanical environment via osteocytes in relation to osteophyte maturation could be one factor driving the following adaptation of osteophytes that involves macro- and micro-architectural changes or can be explained by pain and limited function. Also, the presence of subchondral cysts that was detected in our population, suggests an influence of mechanical changes in the joint that could subsequently lead to alterations in bone remodelling^39,40^.

Differences of the mechanosensitive network of osteocytes could hold key answers to osteophyte adaption. Osteocytes were more numerous in the osteophyte bone compared to the adjacent subchondral bone implying a different network connectivity in osteophytes. However, this observation was not accompanied by changes in number of canaliculi per osteocyte lacuna. The density of osteocyte lacunae within osteophytes ranged from 400–450 lacunae per mm² bone area. We previously reported that young individuals from both sexes (around 20 years of age) have a total amount of osteocyte lacunae per bone area of 350–450 in the periosteal area of femoral cortexes^29,41^. This confirms that the osteophyte bone tissue quality resembles young bone tissue not just regarding mineralization indices but also with respect to osteocyte number. In combination with the irregular collagen fiber arrangement, the lower BMD determined and the large amount of new bone formation, the osteophyte bone consists of woven bone tissue^42^. This newly formed type of bone is generally found in fetal bones^41,43^ and with fracture healing^44,45^ and is later on replaced by a more organized, lamellar type of bone matrix^41,46,47^.

In addition, we determined in this study a lower number of empty lacunae in osteophytes compared to the neighbouring bone tissue. The osteocyte network in OA has been shown to be altered with increased osteocyte cell death^48,49^ and network connectivity. With a net higher number of viable osteocytes and lower empty lacunae as seen in OA osteophytes of our study, OA-dependent osteocyte network disruption seems locally improved. This could result in improved mechanical strain signal transduction and be the cause of the detected high bone turnover. In addition, we determined slight changes in osteocyte network characteristics in relation to the maturation stages of osteophytes. The apparent differences in osteocytes’ morphology and orientation between early and late stage osteophyte are in agreement with differences in terms of the osteocyte-lacunar properties reported for woven and lamellar bone^50–53^. While the differences in 2D morphology and orientation did not reach statistical significance, due to the large number of osteocytes per 3D bone volume, their total sum is much greater. The variations in osteocyte morphology and orientation could reflect an adaptation of the network to differences in the micromechanical environment and matrix strain^54–56^; affecting osteocyte mechanosensation and –transduction^57,58^ in maturing osteophytes.

The study has a few limitations. The sample material was obtained exclusively from OA patients. Thus, osteophytes from healthy control individuals were not analysed due to ethical reasons. The functional (i.e. biomechanical) role of osteophytes in joint re-stabilisation was not assessed in the current study design. It would be worthwhile to correlate the formation of osteophytes in the knee OA with functional biomechanical parameters of the knee joint to further evaluate how osteophyte formation could affect mechanical joint stabilization. Furthermore, the quantification of the osteocyte network has been performed in a 2D approach. While quantitative two-dimensional histological and immunohistochemistry imaging can provide valuable information on mechanistic cellular properties, a quantification of the osteocyte lacuno-canalicular network in 3D visualization would provide further valuable information. Lastly, the effect of hormones on osteophyte formation and maturation was not assessed. Since hormonal factors could affect bone remodelling^59^, investigation of hormones would provide further insight into their effect on osteophyte adaptation process.

In conclusion, we found within tibial OA osteophytes a lower mineral density and an active remodelling process, with persistent microarchitectural changes, accompanied with more numerous osteocytes, reinforcing the notion that osteophytes develop in progressively altering OA joints. Our study provides new data on the structural characteristics of osteophytes suggesting cellular and architectural changes that are related to maturation. Osteophyte characteristics change throughout maturation stages with a transition from woven to lamellar bone accompanied by distinct osteocyte network characteristics and bone turnover rates that result in bone matrix alteration in individual osteophytes. Future studies are needed to prove the intriguing hypothesis that osteophytes can form and grow to adapt to the altering mechanical environment of the previously instable OA joint.

## Methods

### Subjects and specimens

Twenty-three patients with knee osteoarthritis that were scheduled for a knee arthroplasty were selected for this study. The mean age of the patients was 65 ± 9 years, with 7 male and 16 female patients. In the tibial plateaus collected, a total of 43 osteophytes were identified.

A full-size tibia plateau section of about 2–3 cm thickness was taken from each patient and immediately placed into 4% neutrally-buffered paraformaldehyde solution. Inclusion criterion was the presence of osteophytes as noted by the orthopaedic surgeon. Patients with bone necrosis, joint infections and rheumatoid arthritis were excluded. Written informed consent to donate the tibia plateau as surgical waste material was received from all patients. Specimens were analysed in an anonymized fashion as approved by the local ethics committee (Hamburg Chamber of Physicians, WF-020/17). All experiments were performed in accordance with local guidelines and regulations.

### Macroscopic specimen characterization

Bright field images of the joint surface and the osteophyte appearance in the tibia plateau specimens were taken using a KAISER Scando dyn A+ camera (Germany) (Fig. 1a). Further macroscopic analysis involved the radiographic examination using a Faxitron X-ray cabinet, which allowed identification of osteophytes, including a 2D representation of the bone structure in each specimen (Fig. 1b).

### Microscopic characterization using microcomputed tomography

High-resolution peripheral quantitative computed tomography scans (HR-pQCT) was performed using an XtremeCT (Scanco Medical AG®, Brüttisellen, Switzerland). The global bone microstructure of each specimen was assessed using a nominal isotropic voxel size of 82 μm and using the standard *in vivo* scanning protocol (60 kVp, 900 μA) as previously described^23^. A 3D reconstruction of the bone matrix structure was performed using the Scanco software (Fig. 1c,d).

Osteophytes were then segmented from sections using a diamond band saw (EXAKT, Norderstedt, Germany). To this end, osteophytes and the adjacent subchondral bone were sawed from each specimen (1–3 osteophytes per patient). In addition, a bone section distant from osteophytes was taken from the same side of the tibia plateau to be used as control subchondral bone (Fig. 3). These samples were then scanned using a µCT 40 (Scanco Medical, Brüttisellen, Switzerland) with an isotropic voxel size of 15 µm at 55 kV, 145 µA and 200 ms integration time.

The 3D microarchitecture of the trabecular bone was determined in the µCT scans using a global bone threshold of 400 mg HA/cm^3^. The structural comparison was possible in 22 matched observations, with one patient not having enough control area to allow for analysis. The following parameters were determined: tissue volume (TV); bone volume (BV/TV); trabecular thickness (Tb.Th); trabecular separation (Tb.Sp); trabecular number (Tb.N); Structure Model Index (SMI); connectivity density (Conn.D) degree of anisotropy (DA) and bone mineral density (BMD, calibrated with hydroxyapatite standards) using the Scanco software.

### Histological sample preparation and qualitative evaluation

For histology, samples were cut in half using a band saw (EXAKT, Norderstedt, Germany). One half was prepared for undecalcified histology and the other for decalcification and analysis after complete demineralization. Both parts were dehydrated in ascending concentrations of ethanol. The first half was further processed for polymethylmethacrylate (PMMA) embedding, consecutive sections of 4 µm-thickness were cut using a Leica microtome (Wetzlar, Germany) and toluidine blue staining was performed to assess bone cells and bone matrix. The other part of each sample was decalcified in 20% EDTA (neutrally buffered) and embedded in paraffin. Sections of 4 µm-thickness were stained with hematoxylin and eosin (H&E), safranin O, Picrosirius red and Ploton silver precipitation^24^. H&E staining helped to determine the presence of a fibrous surface tissue layer. Safranin O was used to assess the presence and quality of the cartilaginous matrix. To provide a qualitative analysis of the lamellar structure of type I collagen, sections stained with Picrosirius red were evaluated with circularly polarized light microscopy (BX63, Olympus, Germany). TRAP activity staining determined bone resorbing osteoclasts. Sclerostin immunohistochemistry detected sclerostin-positive osteocytes.

### Quantitative characterization of the osteocyte network

Quantifications of the osteocyte network within the samples were performed on 10 randomly-selected osteophytes and their corresponding subchondral bone. All analyses were performed manually in a blinded fashion using the Fiji software (ImageJ 1.51 k, Wayne Rasband, National Institute of Health, USA). The osteocyte lacunar distribution was evaluated on H&E stained sections through the following parameters: number of lacunae with osteocytes (Ot.N) and the number of empty lacunae (e.Lac.N). These parameters were evaluated in four random squares within the trabecular bone (1.51 mm^2^) of the osteophyte and the subchondral bone. The lacunar size and number of canaliculi per lacunae were evaluated in sections labelled with silver precipitation. The mean lacunae size was determined in about 50 cells per sample. The canaliculi number per lacuna was determined considering canaliculi connected with the lacuna in the plane of view. On average 12 osteocytes per area were evaluated.

### Statistical analysis

Results were expressed as mean ± standard deviation. Data were analysed using GraphPad Prism 5 software (La Jolla, California, USA). Data distribution was assessed by Kolmogorov-Smirnov test. Parametric tests were performed due to normal data distribution. The µCT data comparing osteophyte, subchondral bone and control area was assessed by one-way ANOVA using a Tukey post-hoc test with *p < 0.05 and (a > b). The µCT data comparing the osteophyte maturation stages were performed by unpaired t-test with *p < 0.05. The osteocyte network data was analysed using a paired t test.

### Ethical approval

Hamburg Chamber of Physicians - WF-020/17.

## Supplementary information

Supplemental figures 1 and 2.
