# Supplementary material for: Multiscale bone quality analysis in osteoarthritic knee joints reveal a role of the mechanosensory osteocyte network in osteophytes
Source: Sci Rep. 2020 Jan 20;10:673. doi: 10.1038/s41598-019-57303-z (PMC6971279; doi:10.1038/s41598-019-57303-z)

SUPPLEMENTAL INFORMATION

Multiscale bone quality analysis in osteoarthritic knee joints reveal a role of the mechanosensory osteocyte network in osteophytes

Gustavo Davi Rabelo[1*], Annika vom Scheidt[1*], Felix Klebig[2], Haniyeh Hemmatian[1], Mustafa Citak[2], Michael Amling[1], Björn Busse[1], Katharina Jähn[1*]

[1] Heisenberg Research Group, Department of Osteology and Biomechanics, University Medical Center Hamburg-Eppendorf, Hamburg, Germany

[2] Helios-ENDO Klinik Hamburg, Hamburg, Germany

*: Equal contribution to the manuscript

SUPPLEMENTAL FIGURE LEGENDS

Supplemental figure 1: Osteophyte features. a-c) Various thickness of a fibrous tissue layer (white double headed arrows) on top of the osteophyte surface (decalcified, Safranin O, fastgreen) and a thick cartilage layer seen in b) (*). d) Cartilage of a control area, e) cartilage on top of an osteophyte revealing cracks and fissures (white arrow) and cell clusters (*) (decalcified, H&E).

Supplemental figure 2: Histological characteristics of the osteocyte network. a) Osteophyte bone revealing lacunae with single osteocyte and two nuclei (red arrow; decalcified, H&E, inserts magnified 1.8x). b) Osteocyte lacunar outline (white arrows) and individual canaliculi emerging from one lacuna (black arrows; decalcified, silver precipitation).

SUPPLEMENTAL FIGURES

Supplemental Figure 1:

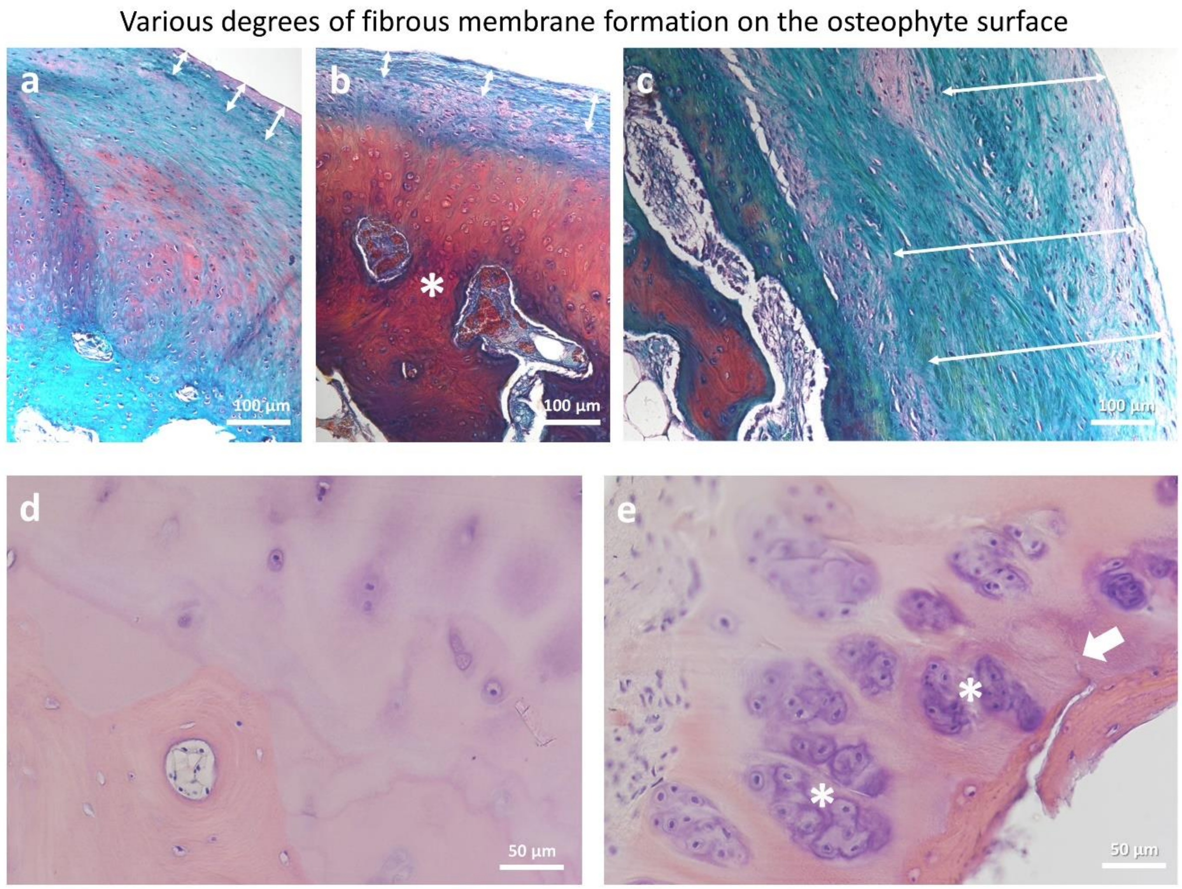

Supplemental Figure 2:

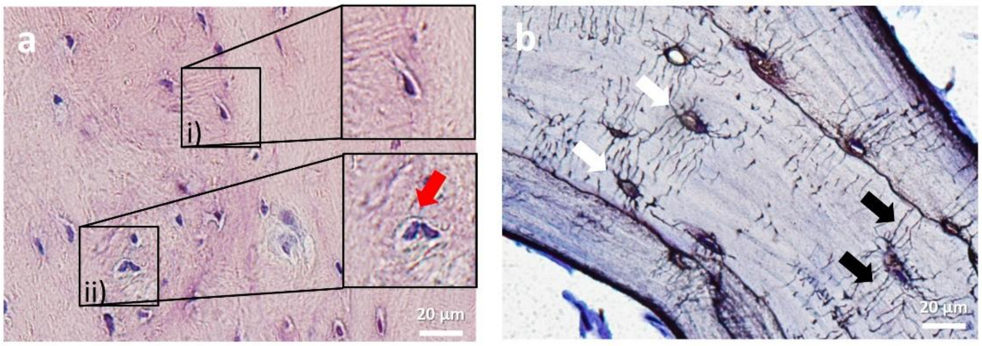

Supplement: Supplementary file 1 — Supplemental figures 1 and 2. [file 41598_2019_57303_MOESM1_ESM.pdf]
